# Development of a Viral RdRp-Assisted Gene Silencing System and Its Application in the Identification of Host Factors of Plant (+)RNA Virus

**DOI:** 10.3389/fmicb.2021.682921

**Published:** 2021-07-29

**Authors:** Wang Zhang, Yanglin Qiu, Lingyun Zhou, Jinlong Yin, Liqun Wang, Haijian Zhi, Kai Xu

**Affiliations:** ^1^Jiangsu Key Laboratory for Microbes and Functional Genomics, College of Life Sciences, Nanjing Normal University, Nanjing, China; ^2^National Center for Soybean Improvement, National Key Laboratory for Crop Genetics and Germplasm Enhancement, Key Laboratory of Biology and Genetic Improvement of Soybean-Ministry of Agriculture, Nanjing Agricultural University, Nanjing, China

**Keywords:** dsRNA, viral RdRp, gene silencing, SAR1, (+)RNA virus

## Abstract

Gene silencing induced by hairpin RNA or virus infection expression is one of the major tools in genetics studies in plants. However, when dealing with essential genes, virus-induced gene silencing (VIGS) and transgenic expression of hairpin RNA could lead to plant death, while transient expression of hairpin RNA in leaves is often less competent in downregulating target gene mRNA levels. Here, we developed a transient double-stranded RNA (dsRNA) expression system assisted by a modified viral RNA-dependent RNA polymerase (RdRp) in plant leaves. We show that this system is more effective in inducing gene silencing than the intron-spliced hairpin RNA expression. Furthermore, by using this system, we tested the role of the early secretory pathway during infection of *Soybean mosaic potyvirus* (SMV). We found that key components of the coat protein complex II vesicles are required for the multiplication of SMV. Overall, this dsRNA-based gene silencing system is effective in downregulating plant gene expression and can be used to identify host genes involved in plant-virus interactions.

## Introduction

Gene silencing is a cellular mechanism that acts on both transcriptional and posttranscriptional levels to regulate gene expression. Double-stranded RNA (dsRNA) is the key trigger of gene silencing and can be artificially delivered by many vector-based systems (Wesley et al., 2001; Schwab et al., 2006; Senthil-Kumar and Mysore, 2014). In plants, dsRNA in the form of an intron-spliced hairpin RNA (ihpRNA) was demonstrated to be effective in inducing the posttranscriptional gene silencing (PTGS), comparing with the single-stranded sense or antisense RNA fragments or the hairpin RNA without intron as the spacer (Smith et al., 2000; Wesley et al., 2001). However, the ihpRNA could not lead to total degradation of target mRNA and often allow detectable levels of target gene activity (Wesley et al., 2001). The ihpRNA transgenic *Arabidopsis* plants targeting, for example, the polyphenol oxidase encoding gene *PPO* or the ethylene signaling gene *EIN2* showed a 70% or 66% silencing rate in the progeny plants (Wesley et al., 2001). A model system based on transient expression of ihpRNA in *Nicotiana benthamiana* leaves showed that the transcripts level of targeted ectopically expressed β-glucuronidase or green fluorescence protein-coding gene only reduced 76% or 64% (Yan et al., 2012). The remaining transcripts of target genes could still be translated and contribute to the phenotype, thus limiting the use of ihpRNA-induced PTGS in genetics studies.

Besides ihpRNA, plant (+)RNA viruses are often used as vectors to deliver dsRNA produced during the viral RNA replication (Liu et al., 2002; Zhang and Ghabrial, 2006; Yuan et al., 2011). The virus-induced gene silencing (VIGS) is a robust way to downregulate the expression of target gene transiently. However, the VIGS system does have limitations. Some rescued viruses could, for example, cause symptoms on the host plants (Ratcliff et al., 2001; Zhang and Ghabrial, 2006; Yuan et al., 2011) or change the metabolism of the infected cells (Fernandez-Calvino et al., 2014), thus could potentially bring complications in the interpretation of the genetic data. Limited viral host range, compatible interaction between the virus and some cultivated varieties, and the regulation on biosafety and environmental release could also potentially hamper the use of VIGS (Purkayastha and Dasgupta, 2009).

In this study, we developed a plant-based virus-free dsRNA delivery system that takes advantage of a cytoplasm-localized viral RNA-dependent RNA polymerase (RdRp) to amplify the single-stranded RNA template into dsRNA. This system does not require a live virus to generate the silencing signal. This viral RdRp-assisted silencing (VRAS) system reduced the mRNA of the target gene to a lower level than the ihpRNA. We further showed that this VRAS system could be used to identify host genes involved in plant-virus interactions.

## Results

### Configuration of the Viral RdRp-Assisted Gene Silencing System

**FIGURE 1:**
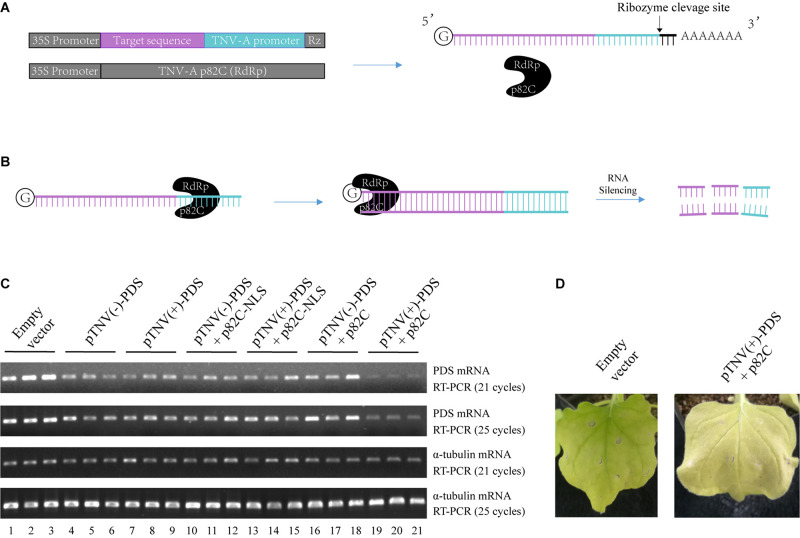
The viral RdRp-assisted gene silencing vectors induce silencing of PDS. **(A)** Scheme of the VRAS vectors. The first vector containing both a 35S promoter and a TNV-A positive strand RNA initiation promoter provides the sense-strand RNA for dsRNA synthesis. The second vector is used for the expression of a truncated form of TNV-A RdRp p82C. **(B)** The p82C RdRp recognizes the TNV-A promoter to initiate synthesis of the complementary strand of the sense-strand RNA, then the dsRNA generated by the VRAS system leads to RNA silencing of the target gene. **(C)** The TNV-A promoters for positive-strand or negative-strand RNA synthesis and p82C proteins targeted to the nucleus or the cytoplasm were tested for their ability in inducing the *PDS* silencing in the VRAS system. Agrobacteria transformed with pCB301-314-pTNV(+)-NbPDS-Rz, pCB301-314-pTNV(−)-NbPDS-Rz, pGD-2 × 35S-L-TNV-p82C, or pGD-2 × 35S-L-TNV-p82C-NLS were mixed as shown and infiltrated into *N. benthamiana* leaves (OD_600_ of 0.5, each). Semiquantitative PCR of 21 or 25 cycles was performed to measure the mRNA levels for PDS or α-tubulin coding genes at 2 dpi. **(D)** Image of the plant leaf silenced for *PDS* gene at 10 dpi. Each experiment was repeated three times.

To circumvent the use of infectious virus but still exploit the ability of viral RdRp in amplifying the RNA template, we have developed a two-vector-based system to initiate the synthesis of dsRNA *in vivo* for the induction of gene silencing. First, we cloned the C-terminal part of the p82 (p82C) RdRp from the tobacco necrosis virus A (TNV-A) Chinese isolate (Xi et al., 2008). The p82 protein is produced by translational readthrough at the amber stop codon of the first ORF. The C-terminal part of p82 includes the amino acids downstream of the amber stop codon and contains the RdRp motif (Xi et al., 2008). TNV-A is a type member of the *Alphanecrovirus* genus in the *Tombusviridae* family. Its replication is initiated by the recognition of viral RdRp to the 3′-end of viral (+) or (−) RNA sequence, also known as the viral promoter (Song and Simon, 1994). We cloned the p82C RdRp under the control of a 35S promoter for plant expression (Figure 1A). The sequence of the gene targeted for silencing was then placed between a 35S promoter and a TNV-A promoter (Figure 1A). A *Hepatitis delta virus* antigenomic ribozyme (Rz) (Ferre-D’Amare et al., 1998) was placed at the downstream end of the TNV-A promoter to ensure the release of the promoter. This configuration of double promoter allows the initial RNA polymerase II (POL II)-mediated synthesis of the target gene sequence and the following TNV-A promoter-driven p82C RdRp-mediated synthesis of the complementary sequence, thus producing plenty of dsRNA for targeted gene silencing (Figure 1B).

### Optimization of the Viral RdRp-Assisted Gene Silencing System

We optimized this system in two aspects: to find the more active viral promoter sequence and to choose the optimal subcellular localization of the viral RdRp. The amount of products made by viral RdRp is affected by the context of the promoter sequences that form different RNA structures (Song and Simon, 1994; Koev et al., 2002; Rajendran et al., 2002; Sun and Simon, 2006). The promoter for positive-strand RNA synthesis is often more active than the promoter for negative-strand RNA synthesis (Koev et al., 2002; Rajendran et al., 2002). We have cloned both positive- and negative-strand initiation promoters. Also, since the sense-strand of the target sequence is synthesized in the nucleus by RNA polymerase II, a p82C fused with nuclear localization sequence (p82C-NLS) was designed to test whether this nucleus-localized version is more efficient in the induction of gene silencing.

A 436-bp sequence from the *Nicotiana benthamiana* phytoene desaturase (PDS) open reading frame was cloned and inserted into the vector containing TNV-A promoter for either positive- or negative-strand viral RNA synthesis [pTNV(+)-PDS or pTNV(−)-PDS]. Each of these two vectors was agroinfiltrated into *N. benthamiana* leaves alone, with p82C-NLS, or with p82C (Figure 1C). After ∼2 days, the total RNAs were extracted from the infiltrated leaves and subjected to first-strand cDNA synthesis using oligo(dT)18 as a primer. The level of PDS mRNA accumulation in the total RNAs was detected by semiquantitative reverse transcription polymerase chain reaction (semiquantitative PCR). The housekeeping gene encoding α-tubulin was used as an internal control. The results showed that coexpression of p82C and mRNA containing *PDS* sequence driven by TNV-A positive-strand initiation promoter greatly reduced the *PDS* mRNA level (Figure 1C, lanes 19–21). In the absence of p82C, the transcription of *PDS* target sequence could only moderately reduce the *PDS* mRNA level (Figure 1C, lanes 4–9). The target gene degradation in the absence of p82C might be due to the ribozyme cleavage that produced a poly(A)-lacking mRNA, which can be recognized by endogenous plant RdRp (RDR6) to trigger gene silencing (Baeg et al., 2017). Moreover, we observed no additional effect for co infiltration of vectors carrying p82C-NLS and the template RNA for the target gene (Figure 1C, lanes 10–15 vs. lanes 4–9), suggesting that the nucleus localized p82C could not induce gene silencing. At 10 days postinfiltration (dpi), the “pTNV(+)-PDS + p82C”-infiltrated leaves display obvious albino phenotype, suggesting successful silencing of the *PDS* gene (Figure 1D).

### Involvement of the RdRp in the dsRNA Synthesis

**FIGURE 2:**
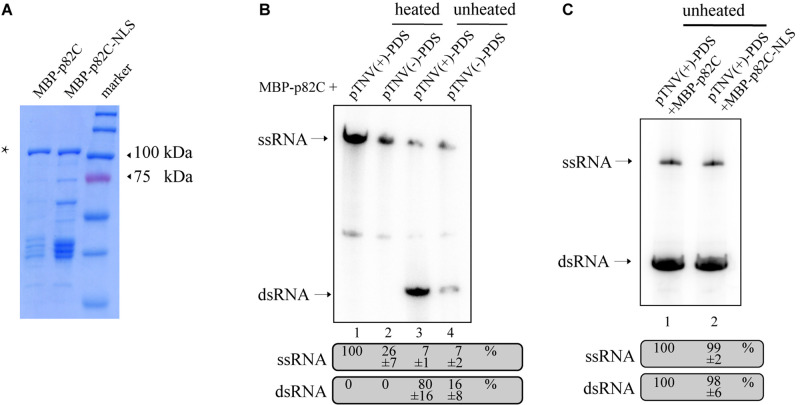
*In vitro* RdRp assay to test the activity of promoters of TNV-A. **(A)** Purified recombinant MBP-p82C and MBP-p82C-NLS analyzed by SDS-PAGE and Coomassie blue staining. The band of MBP-p82C or MBP-p82C-NLS is marked with an asterisk. **(B)** The non-denaturing PAGE analysis of complementary RNA synthesized by MBP-p82C under the control of TNV-A positive- or negative-strand RNA initiation promoters. The ssRNA band of lane 1 was chosen as 100%. The experiment was repeated three times. **(C)** The non-denaturing PAGE analysis of complementary RNA synthesized by MBP-p82C or MBP-p82C-NLS under the control of TNV-A positive-strand RNA initiation promoter. The ssRNA and dsRNA bands of lane 1 were chosen as 100%, respectively. The experiment was repeated three times.

The obtained data also showed that the promoter for positive-strand RNA synthesis [pTNV(+)] is more efficient in inducing *PDS* silencing than the promoter for negative-strand RNA synthesis [pTNV(−)] when coexpressed with p82C (Figure 1C, lanes 19–21 vs. lanes 16–18). We proposed that the positive-strand initiation promoter of TNV-A is more robust in driving dsRNA synthesis. To test this hypothesis, we performed the *in vitro* RNA synthesis assay. An N-terminally maltose-binding protein (MBP) tagged p82C was expressed in *Escherichia coli* and purified by affinity chromatography using amylose resin (Figure 2A). The recombinant protein MBP-p82C was then incubated with *in vitro*-transcribed RNA template containing *PDS* gene fragment followed by either positive or negative promoter of TNV-A. The complementary strand of the RNA template was synthesized by MBP-p82C in the presence of radioactively labeled rNTPs. The resulting products were either heated to denature the dsRNA or unheated to keep the dsRNA formation and then separated by non-denaturing polyacrylamide gel electrophoresis (PAGE) and imaged using a phosphorimager. Upon heat treatment, the dsRNA unwinded, and the radioactively labeled RNA products migrated as single-stranded RNA corresponding to the template size (Figure 2B, heated). The MBP-p82C synthesized ∼4 times more products using a TNV-A positive-strand initiation promoter-driven template (Figure 2B, lane 1 vs. lane 2). While in the unheated samples, most of the newly synthesized ssRNA products hybridized with the template as dsRNA (Figure 2B, unheated). The remaining RNA products kept in ssRNA format were probably due to weak terminal transferase activity copurified with MBP-p82C that can label the template RNA or due to insufficient dsRNA winding (Figure 2B, lanes 3–4, ssRNA band). Nevertheless, ∼5 times more dsRNAs were produced when using the viral positive-strand initiation promoter (Figure 2B, lane 3 vs. lane 4). Overall, the positive-strand initiation promoter supported 4∼5-fold higher complementary RNA synthesis than the promoter for the negative strand. These results demonstrated that the higher silencing efficiency in the “pTNV(+)-PDS + p82C” combination is due to more active promoter activity that leads to more dsRNA production.

Since the p82C-NLS cannot effectively induce *PDS* silencing (Figure 1C), thus we tested whether the nuclear localization sequence (NLS) alone has any effect on the RdRp activity of p82C. We found that the recombinant protein containing the nuclear localization sequence (MBP-p82C-NLS) has comparable *in vitro* RdRp activity with MBP-p82C (Figure 2C), suggesting that the nuclear localization rather than the attachment of NLS caused ineffective *PDS* silencing.

### Comparison of VRAS and ihpRNA-Induced Gene Silencing

**FIGURE 3:**
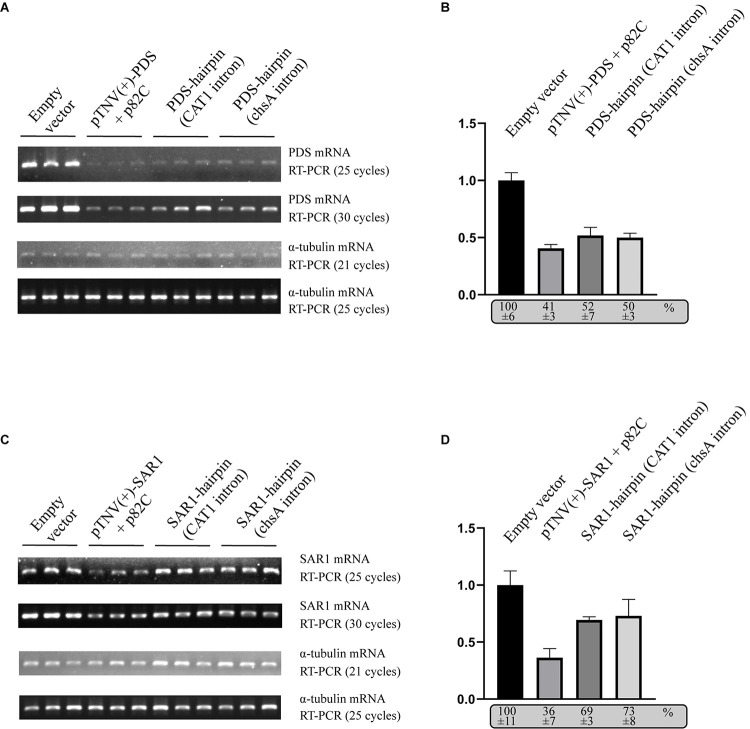
Comparison of the VRAS- and ihpRNA-mediated silencing of *PDS* or *SAR1* genes in *N. benthamiana*. **(A)** Semiquantitative PCR was performed to measure the mRNA levels for PDS or α-tubulin coding genes from leaves agroinfiltrated with empty vector [pCB301-314-pTNV(+)-RZ] (OD_600_ of 1.0), VRAS vectors [pCB301-314-pTNV(+)-NbPDS-Rz and pGD-2 × 35S-L-TNV-p82C] (OD_600_ of 0.5, each), ihpRNA expression vector pGD-35S-CAT1-NbPDS-hairpin (OD_600_ of 1.0), or ihpRNA expression vector pGD-35S-chsA-NbPDS-hairpin (OD_600_ of 1.0) at 3 dpi. PCR products were analyzed by agarose gel electrophoresis. The number of PCR cycles is shown. **(B)** qPCR measuring *PDS* mRNA accumulation. The PDS mRNA level from empty vector-infiltrated leaf samples was set as 100%. Standard error was calculated from three independent repeats. See more details in panel **(A)**. **(C)** Semiquantitative PCR was performed to measure the mRNA levels for PDS or α-tubulin coding gene from leaves agroinfiltrated with empty vector [pCB301-314-pTNV(+)-RZ], VRAS vectors [pCB301-314-pTNV(+)-NbSAR1-Rz and pGD-2 × 35S-L-TNV-p82C], ihpRNA expression vector (pGD-35S-CAT1-NbSAR1-hairpin), or ihpRNA expression vector (pGD-35S-chsA-NbSAR1-hairpin) at 3 dpi. PCR products were analyzed by agarose gel electrophoresis. The number of PCR cycles is shown. **(D)** qPCR measuring *SAR1* mRNA accumulation. See more details in panel **(B)**.

To compare the VRAS system with the intron-spliced hairpin RNA in the efficiency of inducing gene silencing, we constructed two binary vectors that drive the transcription of ihpRNA under the control of a 35S promoter. One contains a 190-bp-long intron of castor bean catalase gene (*CAT1*) (Tanaka et al., 1990). The other vector contains a petunia chalcone synthase A (chsA) intron that is 1,349-bp long in length (Kerschen et al., 2004). The same 436 bp gene fragment of *PDS* used for p82C-mediated silencing was cloned in both sense and antisense orientations on each side of the intron loop to form the typical ihpRNA structure. Each of these *PDS*-ihpRNA expression vectors, the VRAS vectors “pTNV(+)-PDS + p82C,” and the empty vector were agroinfiltrated into *N. benthamiana* leaves. Infiltrated leaves were collected ∼3 days after agroinfiltration and subjected to mRNA analysis using semiquantitative PCR and quantitative PCR (qPCR). The results showed that the PDS mRNA reduced to 41% in the leaf samples infiltrated with the VRAS vectors compared with the empty control at 3 dpi. In the *PDS*-ihpRNA silenced leaf samples, PDS mRNA reduced to 52 or 50%, suggesting that the VRAS system is more efficient in silencing the *PDS* gene (Figures 3A,B).

We also tested the silencing of the *SECRETION-ASSOCIATED RAS 1* (*SAR1*) encoding an Arf family small GTPase. SAR1 regulates protein export at the ER export sites (ERES) and is essential in plant growth and development (Hanton et al., 2008; Liang et al., 2020). The VRAS system reduced the *SAR1* mRNA level to 36% at 3 dpi (Figure 3D), while the *SAR1* mRNA only reduced to 69% or 73% in *SAR1*-ihpRNA-expressed leaves. Thus, we conclude that the VRAS is more robust than the ihpRNA in inducing gene silencing when transiently expressed.

### Using VRAS to Study Plant-Virus Interactions

To test whether VRAS can be applied in genetics studies in plant-virus interactions, we tested the effect of VRAS-mediated *SAR1* silencing on the replication of *Soybean mosaic potyvirus* (SMV). SMV belongs to the *potyvirus* genus, *potyviridae*, the replication of which takes place on the cytoplasmic surface of reorganized intracellular membranes (Wei and Wang, 2008; Cabanillas et al., 2018; Wu et al., 2020). Membrane trafficking of the early secretory system was shown to be required for the replication of several (+)RNA viruses (Belov et al., 2007; Wei and Wang, 2008; Inaba et al., 2019). However, the requirement of SAR1 during SMV replication was not yet demonstrated.

**FIGURE 4:**
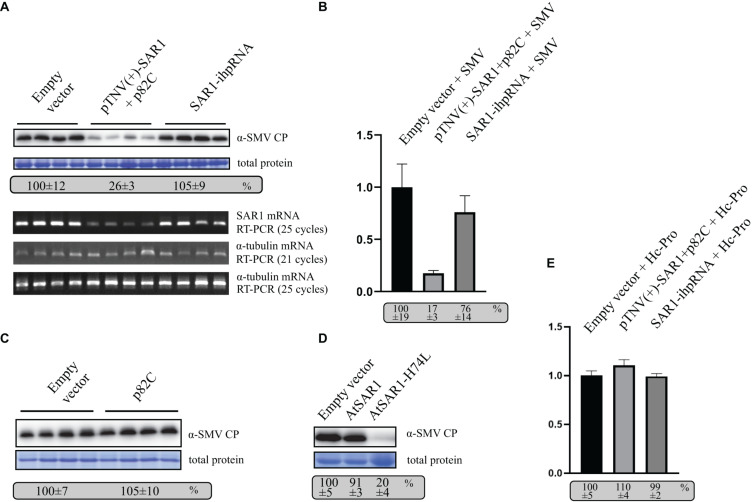
VARS system-induced *SAR1* silencing inhibits SMV replication. **(A)** SMV accumulation in leaf samples infiltrated with empty vector [pCB301-314-pTNV(+)-RZ] (OD_600_ of 0.8) or samples silenced for *SAR1* by VRAS vectors (OD_600_ of 0.4, each) or by ihpRNA-expressing vector (OD_600_ of 0.8) in the presence of SMV infectious clone (OD_600_ of 0.2) was measured by Western blotting at 5 dpi. Total protein loadings were shown as Coomassie blue-stained gel (upper panels). Semiquantitative PCR analysis was performed to measure the mRNA levels of SAR1 or α-tubulin coding gene in the SMV-infected leaves 5 days after viral infection. The number of PCR cycles is shown by each gel (lower panels). **(B)** qPCR measuring *SAR1* mRNA accumulation. The *SAR1* mRNA level from empty vector infiltrated leaf samples was set as 100%. Standard error was calculated from three independent repeats. See more details in panel **(A)**. **(C)** SMV accumulation in leaf samples infected with SMV infectious clone (OD_600_ of 0.2) and coexpressed with p82C (OD_600_ of 0.8) was measured by Western blotting at 5 dpi. Total protein loadings were shown as Coomassie blue-stained gel. **(D)** SMV accumulation in leaf samples infected with SMV infectious clone (OD_600_ of 0.2) and coexpressed with wt AtSAR1 or AtSAR1-H74L dominant-negative mutant (OD_600_ of 0.2) was measured by Western blotting at 5 dpi. Total protein loadings were shown as Coomassie blue-stained gel. **(E)** qPCR measuring *SAR1* mRNA accumulation in leaf samples infiltrated with empty vector [pCB301-314-pTNV(+)-RZ] (OD_600_ of 0.8) or VRAS vectors for *SAR1* silencing (OD_600_ of 0.4, each) or ihpRNA-expressing vector (OD_600_ of 0.8) in the presence of SMV Hc-Pro expression (OD_600_ of 0.2) at 5 dpi. The *SAR1* mRNA level from empty vector and Hc-Pro-infiltrated leaf samples was set as 100%. Standard error was calculated from three independent repeats.

To test the role of SAR1 in SMV replication, VRAS vectors expressing pTNV(+)-SAR1 and p82C were coinfiltrated with the infectious clone of SMV into *N. benthamiana* leaves. Empty vector or ihpRNA containing *SAR1* coding region and chsA intron (SAR1-ihpRNA) were also coinfiltrated with SMV, respectively, as controls. Five days after infiltration, leaf samples were collected for protein and RNA analysis. We found that during SMV replication, *SAR1* mRNA reduced to 17% in VRAS-treated samples at 5 dpi comparing with the empty vector control (Figures 4A,B), while ihpRNA-mediated *SAR1* silencing could only reduce the *SAR1* mRNA to 76%. Accordingly, SMV accumulation reduced to 26% in samples silenced for *SAR1* by VRAS but kept unchanged in samples expressing ihpRNA (Figure 4A). These results demonstrated that SAR1 is required for SMV replication. The unchanged viral replication in SAR1-ihpRNA-infiltrated leaves may be because SAR1 expression contributed by the 76% non-silenced *SAR1* mRNA is enough for the optimal SMV replication (Figure 4B). In addition, we have shown that p82C expression alone does not affect SMV replication (Figure 4C).

Furthermore, we expressed SAR1 dominant-negative (DN) mutant in *N. benthamiana* replicating SMV *via* agrobacterium-mediated transient expression. The SAR1-DN can block the wild-type SAR1 function in regulating the early secretory pathway (Takeuchi et al., 2000). The expression of SAR1-DN (AtSAR1-H74L) led to reduced SMV accumulation (Figure 4D), confirming the essential role of SAR1 in SMV replication.

The potyviral Hc-Pro is a well-documented viral suppressor of gene silencing (Valli et al., 2018). We found that transiently expressed Hc-Pro under the control of 35S promoter strongly inhibited both VRAS- or ihp-RNA-mediated *SAR1* silencing (Figure 4E). In contrast, *SAR1* can be silenced during SMV infection (Figure 4B). It is likely that SMV-driven Hc-Pro expression is much weaker in suppressing gene silencing, thus allowing the induction of gene silencing for the gene function study in SMV replication.

**FIGURE 5:**
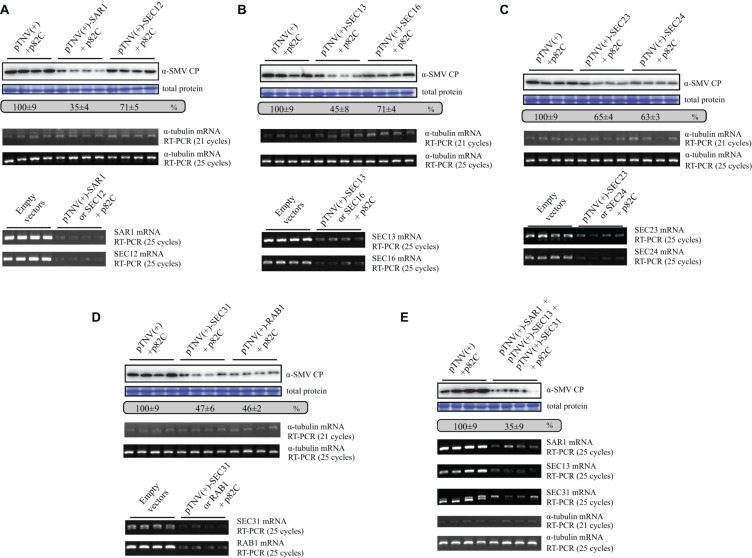
Silencing of genes involved in COPII vesicle forming and biogenesis leads to suppressed SMV replication. **(A)** SMV accumulation in samples infiltrated with SMV infectious clone (OD_600_ of 0.2) and empty VARS vectors (pCB301-314-pTNV(+)-RZ and pGD-2 × 35S-L-TNV-p82C) (OD_600_ of 0.4, each) or VARS vectors for *SAR1* or *SEC12* silencing (OD_600_ of 0.4, each) was measured by Western blotting at 5 dpi. Total protein loadings were shown as Coomassie blue-stained gel (upper panels). Semiquantitative PCR analysis was performed to measure the mRNA levels of α-tubulin coding gene (middle panels), *SAR1*, or *SEC12* (lower panels) in the SMV-infected leaves 5 days after viral infection. The number of PCR cycles is shown. **(B)** SMV accumulation in leaves silenced for *SEC13* or *SEC16*. See further details in panel **(A)**. **(C)** SMV accumulation in leaves silenced for *SEC23* or *SEC24*. See further details in panel **(A)**. **(D)** SMV accumulation in leaves silenced for *SEC31* or *RAB1*. See further details in panel **(A)**. **(E)** SMV accumulation in leaves silenced for *SAR1*, *SEC13*, and *SEC31*. Agrobacteria transformed with vectors expressing pTNV(+)-SAR1, pTNV(+)-SEC13, or pTNV(+)-SEC31 were combined (OD_600_ of 0.16, each) and coinfiltrated with pGD-2 × 35S-L-TNV-p82C (OD_600_ of 0.4) and SMV infectious clone (OD_600_ of 0.2). See further details in panel **(A)**.

Since SAR1 is required for the formation of coat protein complex II (COPII)-coated vesicles, other COPII components or proteins that regulate COPII vesicle transport might also affect SMV replication. To test this hypothesis, genes encoding guanine-nucleotide exchange factor (SEC12), inner-coat of COPII (SEC23 and SEC24), the outer-coat of COPII (SEC13 and SEC31), a peripheral membrane residing scaffold protein required for COPII vesicle formation (SEC16), or Ras-related protein RAB1 GTPase that regulate COPII to Golgi transport (Takeuchi et al., 2000) were silenced by using the VRAS vectors in the presence of SMV infection. SMV accumulation reduced to less than 50% in the leaf tissues silenced for *SEC13*, *SEC31*, and *RAB1* (Figures 5B,D). Silencing of *SEC12*, *SEC16*, *SEC23*, and *SEC24* also reduced SMV accumulation to ∼65–70% (Figures 5A–C). Semiquantitative PCR analysis revealed that the target genes encoding for the COPII components were successfully silenced by the VRAS vectors, while the level of the α-tubulin coding gene was not changed (Figure 5, lower panels). Simultaneous silencing of *SAR1*, *SEC13*, and *SEC31* also reduced SMV accumulation to ∼35%, similar to the level of *SAR1* silencing (Figure 5E). These results demonstrated that the COPII components are required for efficient SMV infection, and the VRAS vectors can be used for the genetics studies of virus-plant interactions.

## Discussion

The ihpRNA vectors are among the most popular tools in genetics study. The ihpRNA is transcribed into pre-mRNA under the control of a promoter and then subsequently spliced into an intron-less hairpin RNA in the nucleus. The splicing event was thought to help align the arms of the hairpin RNA into the dsRNA form, thus improving the efficiency in gene silencing (Smith et al., 2000). Alternatively, splicing was thought to create a loop-less duplex RNA that is probably more nuclease-resistant and stable (Smith et al., 2000). However, a later study used a hpRNA vector containing a spacer region plus an intron between the arms and showed similar silencing efficiency compared with regular intron-containing ihpRNA, which disagreed with the earlier hypotheses (Wesley et al., 2001). Despite the debatable reason, the intron-splicing event undeniably plays a positive role in inducing gene silencing.

In this study, we developed a VRAS system. The VRAS vectors were demonstrated to be more robust in achieving a higher degree of silencing than the ihpRNA (Figures 3, 4). We propose that the VRAS system is advantageous due to the following reasons. In contrast to ihpRNA that forms the RNA duplex in the nucleus, the TNV-A p82C RdRp produces the dsRNA in the cytoplasm. Accordingly, we also observed that the nucleus-located p82C-NLS was inefficiently inducing gene silencing (Figure 1C). The transport of mRNA in the nucleus requires association of RNA binding or modifying proteins during the pre-mRNA processing and consumes ATP (Vargas et al., 2005). It is possible that nuclear export of hairpin structure containing pre-mRNA or dsRNA is the rate-limiting step, and only the exported RNAs can be subjected to the dicer cleavage during the induction of gene silencing.

Although ihpRNA can be successfully applied to investigate gene functions in plant development, it was less frequently used for genetics studies in virus-plant interactions comparing with other methods, especially when transiently delivered (e.g., VIGS or transient expression of dominant-negative mutant). Our study found that the transient expression of ihpRNA cannot sufficiently induce silencing of *SAR1* during SMV infection. In contrast, transient expression of VRAS-based *SAR1* silencing vectors or a SAR1 dominant-negative mutant (H74L) led to inhibited SMV replication and showed advantages over the ihpRNA-based method. The employment of VRAS is currently demonstrated by using the agrobacterium-mediated transient expression assay, which could only be done in a limited number of plants. Further exploration on the stable genetic transformation of VRAS components would expand the application of this system to more plant species.

During the COPII vesicle budding process, GDP-bound SAR1 is activated by its guanine nucleotide exchange factor (GEF) SEC12 into GTP-bound form and becomes membrane associated at ERES (Barlowe and Schekman, 1993). SAR1 forms complex with SEC23 and SEC24 to attract cargo proteins and then recruits SEC13 and SEC31 to form the COPII coat in order to bud from ER (D’Arcangelo et al., 2013). Rab1 GTPase labels the uncoated COPII vesicles and facilitates the Golgi targeting (Westrate et al., 2020). A previous study showed that SAR1 is required to form *Tobacco etch potyvirus* (TEV) 6K2-positive replication vesicles, and expression of SAR1 dominant-negative mutant inhibited TEV infection (Wei and Wang, 2008). Furthermore, SEC23 and SEC24 can colocalize with TEV 6K2 replication vesicles (Wei and Wang, 2008), and SEC24 interacts with *Turnip mosaic potyvirus* (TuMV) 6K2 to facilitate systemic viral movement (Jiang et al., 2015). However, whether SEC23, SEC24, or other COPII components are required for potyvirus replication had not been measured. Based on our genetic study using VRAS-based gene silencing, we found that these COPII components are required for optimal SMV replication. Overall, our work demonstrated that the VRAS system described is a valuable tool in inducing gene silencing and can be applied to the genetics study of virus-plant interactions.

## Materials and Methods

### VRAS and ihpRNA Constructs

To generate the expression vector that contains a 35S promoter and a TNV-A promoter for positive-strand RNA synthesis, the TNV-A positive-strand initiating promoter sequence was first amplified by polymerase chain reaction (PCR) with primer pairs 652/653 (Table 1) by using the infectious clone of TNV-A Chinese isolate pMTC27 (Xi et al., 2008). The resulting PCR product containing the viral promoter and multicloning sites (MCS) was again amplified with primer pair 650/11 in order to add the 5′ homology arm with the pCB301-304 sequence (Sun et al., 2017) as well as the *Hepatitis delta virus* antigenomic ribozyme (Rz) sequence (Ferre-D’Amare et al., 1998). Another round of PCR was performed to extend the 5′ homology arm to the pCB301-304 vector and to add the 3′ homology arm with the pCB301-304 sequence by using primer pair 44/12. The PCR fragment containing the 5′ homology arm, MCS, TNV positive-strand initiating promoter, ribozyme, and the 3′ homology arm was recombined with *Stu*I/*Sma*I-digested pCB301-304 in yeast strain W303-1B (MATα, *leu2-3,112 trp1-1 can1-100 ura3-1 ade2-1 his3-11,15*) through homologous recombination, resulting in pCB301-314-pTNV(+)-RZ. The expression vector containing the TNV-A negative-strand initiating promoter pCB301-314-pTNV(−)-RZ was constructed with the same method except that the primer pair 651/75, instead of 652/653, was used in the first round of PCR. To generate pCB301-314-pTNV(+)-NbPDS-Rz or pCB301-314-pTNV(−)-NbPDS-Rz, the 436-bp sequence from gene coding for phytoene desaturase was amplified from *N. benthamiana* cDNA with primer pair 715/716 using PCR, digested with *Stu*I/*Sal*I, and inserted into *Stu*I/*Sal*I digested pCB301-314-pTNV(+)-Rz or pCB301-314-pTNV(−)-Rz.

**TABLE 1:** Primers used in this study.

Number	Sequence
11	GCTCTCCCTGACCCAGTGGCTCTCCCTTAGCCATCCGAGTGGACGTGCGTCCTCCTTCGGATGCCCAGGTCGGACCGCGAGGAGGTGGAGATGCCATGC
12	ACCGGCAACAGGATTCAATCTTAAGAAACTTTATTGCCAAATGTTTGAACGATCGGGGAAATTCGAGCTCTCCCTGACCCAG
39	GGGGTGGGGCAAAAGCCCCTC
44	GACGTAAGGGATGACGCACAATCCCACTATCCTTCGCAAGACCCTTCCTCTATATAAGGAAGTTCATTTCATTTGGAGAGG
75	GGAGGTGGAGATGCCATGCCGACCCGGGGTGGGGCAAAAGCCC
568	CGCCATATGCACCATCATCATCATCATGGGTGCCTTGAAAGGAGGCCAGG
569	ACGCGTCGACTCAGTTTGATAATCCATTGATCAAATGCTCTCC
607	CGCGGATCCATGGCTCCTAAGAAGAAGAGAAAGGTTCACCATCATCATCATCATGGG
609	CGCGGATCCATGCACCATCATCATCATCATGGG
649	CGCGAGCTCGCACCATCATCATCATCATGGGTGCCTTGAAAGGAGGCCAGG
650	AAGTTCATTTCATTTGGAGAGGCCTGACCTGCAGGTCGACTCTAGAGGATCCCCGGGCCC
651	CTAGAGGATCCCCGGGCCCGGTACCACGCGTATCGATGACAGCCATTGCTATCCAGTTGG
652	CTAGAGGATCCCCGGGCCCGGTACCACGCGTATCGATCGTTGCCGTGTACGTGGGCTGTG
653	GGAGGTGGAGATGCCATGCCGACCCAGTATTCATACCAAGAATACCGGATAGGTGC
715	GAAAGGCCTAGCTCGAGGTCTTCGTTGGGAAC
716	ACGCGTCGACAGAATATGTGCAACCCAGTCTCGTACC
949	AGTATTCATACCAAGAATACCGGATAGGTGC
974	GTAATACGACTCACTATAGGGAGCTCGAGGTCTTCGTTGGGAAC
1042	CGCGGATCCGAGAGATTGGTTCAACATCAGCCAACAC
1044	CGCACGCGTCCCATTTTGCGGACTATGCTGCACATGAATACCTC
1045	ATGTTCTTGGTAGATTGGTTCTATGG
1109	CATGCCATGGGGATCCCCTAGGCTCGAGGAATTCGTAAATTTCTAGTTTTTCTCCTTCATTTTCTTG
1110	CCGGAGCTCGGTCACCTCTAGACTGCAGGTCGACCAATTGCTGTAACTATCATCATCATCATAGAC
1111	CATGCCATGGGGATCCCCTAGGCTCGAGGAATTCGTAAGAATTTCTTATGTTACATTATTACATTCAACG
1112	CCGGAGCTCGGTCACCTCTAGACTGCAGGTCGACCAATTGCTGCAAATTGACCAAAAAAGATGTG
1113	CTAGTCTAGAAGCTCGAGGTCTTCGTTGGGAAC
1114	CCGGAATTCAGAATATGTGCAACCCAGTCTCGTACC
1272	AAAACTGCAGAGAATATGTGCAACCCAGTCTCGTACC
1480	TGCAGGTCGACTCTAGAGTGGAGAAGGGAATAGCGGTATTCGTAACG
1481	ACACGGCAACGATCGATAGCATGAGCATTAGCCTCATCAAGACTAATTTG
1482	CTTCCACCTTGATCTCGCATCG
1483	ATTTCATGGGCTCCTCATGAACTTGG
1484	TGCAGGTCGACTCTAGAGCACACTGCTAGGTCAGATGG
1485	ACACGGCAACGATCGATAACCCTCCAAACAGGAGTCTTGAAG
1486	TGCAGGTCGACTCTAGAGAGCAGGATCACCTTTGCGGAC
1487	ACACGGCAACGATCGATACAAATGTGTGCAGCAACAATATCAC
1488	CTCAGTCCTCTGAATAGCCTCTGG
1489	TGCAGGTCGACTCTAGAGACTGCCCAGTCACCAATTTTTAGTCC
1490	ACACGGCAACGATCGATACACCTTGTGAGCCGAGAAGAGAAC
1491	CATATGCTCCACAATTATGGCAACGATG
1492	TGCAGGTCGACTCTAGAGCAAATGTTCATCAGGCATCCATACCATC
1493	ACACGGCAACGATCGATAAGAATCAAGGCCGGAATCAAAGGATC
1494	TGGCAAGAGGACAAACAATTGCTCC
1495	TGCAGGTCGACTCTAGAGGATGGGGATGCAAGGACAAAATTGC
1496	ACACGGCAACGATCGATACAACCCTGAGGTAAGAAGACTGACTCG
1497	GTGCAAAGGAGTGCAAGTGTTTCC
1498	TGCAGGTCGACTCTAGAGGATTCTGGTGTTGGCAAATCATGTC
1499	ACACGGCAACGATCGATAGCCGATCTCATCAGCAAAAGCC
1654	CGCGCAGACTAATTCGAGCTCGGCTCCTAAGAAGAAGAGAAAGGTTCAC
1655	AGGGAATTCGGATCCGTCGACTCAGTTTGATAATCCATTGATCAAATGCTC

To generate the pGD-2 × 35S-L-TNV-p82C or pGD-2 × 35S-L-TNV-p82C-NLS for expression of p82C or p82C-NLS in plants, the TNV-A p82C sequence was amplified with primer pair 568/569 from pMTC27 using PCR. The resulting sequence was amplified with primer pair 609/569 to generate p82C fragment or with primer pair 607/569 to generate the p82C-NLS fragment that contains the nuclear localization signal (NLS) derived from SV40 large T-antigen (Kalderon et al., 1984). The p82C or p82C-NLS DNA fragment was digested with *Bam*HI/*Sal*I, and then separately inserted into *Bam*HI/*Sal*I digested pGD-2 × 35S-L (Nawaz-ul-Rehman et al., 2016), generating pGD-2 × 35S-L-TNV-p82C or pGD-2 × 35S-L-TNV-p82C-NLS.

To generate the ihpRNA expressing vector pGD-35S-CAT1, the castor bean catalase gene cat1 intron (Hajdukiewicz et al., 1994) was amplified with primer pair 1109/1110 by using pCambia1301 as a template *via* PCR. The resulting PCR fragments were digested with *Bam*HI/*Sac*I and inserted into pGD-35S digested with the same enzyme. To generate the ihpRNA expressing vector pGD-35S-chsA, the petunia chalcone synthase A (chsA) intron sequence was artificial synthesized (General Biosystems Co., Ltd., China), amplified using PCR with primer pair 1111/1112, digested with *Bam*HI/*Sac*I, and inserted into pGD-35S digested with the same enzyme.

To construct the ihpRNA-containing plasmids, the 436-bp sequence from the gene coding for *PDS* was amplified from the *N. benthamiana* cDNA using PCR with primer pair 1113/1114. The resulting PCR product was digested with *Xba*I/*Eco*RI and inserted into *Avr*II/*Eco*RI-digested pGD-35S-CAT1 or pGD-35S-chsA, respectively. The resulting plasmids were digested with *Pst*I/*Xba*I and ligated with PDS gene fragment that was amplified with primer pair 1113/1272 using PCR, resulting in pGD-35S-CAT1-NbPDS-hairpin or pGD-35S-chsA-NbPDS-hairpin.

To construct the VRAS vectors that used for silencing genes involved in COPII vesicles biogenesis, ∼400–500 bp gene fragments from the *SEC12*, *SEC13*, *SEC16*, *SEC23*, *SEC24*, *SEC31*, or *RAB1* were amplified with primer pairs 1480/1481, 1484/1485, 1486/1487, 1489/1490, 1492/1493, 1495/1496, or 1498/1499 from *N. benthamiana* cDNA using PCR and inserted into *Stu*I/*Mlu*I-digested pCB301-304-pTNV(+)-Rz by *in vitro* homologous recombination using the ClonExpress II One Step Cloning Kit (Vazyme, Cat. # C112-01), resulting in pCB301-314-pTNV(+)-NbSEC12-Rz, pCB301-314-pTNV(+)-NbSEC13-Rz, pCB301-314-pTNV(+)-NbSEC16-Rz, pCB301-314-pTNV(+)-NbSEC23-Rz, pCB301-314-pTNV(+)-NbSEC24-Rz, pCB301-314-pTNV(+)-NbSEC31-Rz, and pCB301-314-pTNV(+)-NbRAB1-Rz. The 413-bp gene fragment of the *SAR1* coding region was amplified from *N. benthamiana* cDNA using PCR with primer pair 1042/1044, digested with *Bam*HI/*Mlu*I, and inserted into *Bam*HI/*Mlu*I-digested pCB301-314-pTNV(+)-Rz, generating pCB301-314-pTNV(+)-NbSAR1-Rz.

To generate the *Escherichia coli* expression construct pMAL-c5X-TNV-p82C, the gene fragment of p82C was amplified using PCR with primer pair 649/569, digested with *Sac*I/*Sal*I, and inserted into pMAL-c5X vector (New England Biolabs, Cat. # N8108) digested with the same enzymes. To generate the *Escherichia coli* expression construct pMAL-c5X-TNV-p82C-NLS, the gene fragment of p82C-NLS was amplified from pGD-2 × 35S-L-TNV-p82C-NLS with primer pair 1654/1655 and inserted into *Sac*I/*Sal*I-digested pMAL-c5X by *in vitro* homologous recombination using the ClonExpress II One Step Cloning Kit (Vazyme, Cat. # C112-01).

### Agroinfiltration of *Nicotiana benthamiana* Leaves

Overnight cultures of the *Agrobacterium* strain EHA105 carrying appropriate vectors grown in a 29°C incubator were collected by centrifugation at 4,500 rpm for 15 min. The collected cells were resuspended in MMA media (10 mM MgCl_2_, 10 mM MES, and 200 μM acetosyringone) to reach an optical density measured at a wavelength of 600 nm (OD_600_) of 1.0. The cultures were mixed according to the requirement of each experiment and infiltrated to the underside of leaves of 4-week-old *N. benthamiana* plants using a 1-ml syringe without a needle.

### RNA Extraction and Reverse Transcription Polymerase Chain Reaction

The total RNAs were extracted from *N. benthamiana* leaves. The leaf disks were grounded to fine powders in a 1.5-ml tube with a pestle after freezing in liquid nitrogen. Then 400 μl RNA extraction buffer (0.1 M glycine, 0.1 M NaCl, 0.1 M EDTA pH 8.0, 1% SDS) and 400 μl RNA phenol were added into the tube, briefly mixed, and centrifuged at 15,000 rpm for 10 min at 4°C. About 300 μl upper aqueous phase was transferred into a 1.5-ml tube containing 300 μl water-saturated phenol-chloroform (1:1), mixed thoroughly, and centrifuged at 15,000 rpm for 10 min at 4°C. Then, 200 μl upper aqueous phase was transferred into a 1.5-ml tube containing 500 μl 100% ethanol with 2% (V/V) 3M sodium acetate (pH 5.6). The precipitated total RNA was subjected to centrifugation at 15,000 rpm for 30 min at 4°C, washed with 70% ethanol, and then dissolved in 40 μl dH_2_O. The concentration of the total RNA was measured with an ultraviolet spectrophotometer and then adjusted to 250 ng/μl. About 1 μg RNA was subjected to reverse transcription with oligo(dT)18 by using the M-MLV RTase (Takara, Code No. 639575) followed by semiquantitative PCR analysis. The cDNA was also subjected to qPCR analysis using the ChamQ SYBR Color qPCR Master Mix (Vazyme, Cat. # Q411-02). Relative gene expression levels were calculated using the 2^–ΔΔCt^ method. Primer pairs 1045/1044, 1480/1482, 1483/1485, 1486/1488, 1489/1491, 1492/1494, and 1495/1497 were used for detection of the mRNA levels of *SAR1*, *SEC12*, *SEC13*, *SEC16*, *SEC23*, *SEC24*, and *SEC31*, respectively.

### Protein Purification From *Escherichia coli*

Purification of the MBP-tagged recombinant protein was described previously (Xu et al., 2014). Briefly, the MBP-p82C or MBP-p82C-NLS was expressed in *E. coli* strain BL21 (DE3) transformed with pMAL-c5X-TNV-p82C or pMAL-c5X-TNV-p82C-NLS plasmid. The *E. coli* cells were lysed by sonication, affinity-purified *via* amylose column into a low-salt column buffer (20 mM Tris–HCl pH 8.0, 25 mM NaCl, 1 mM EDTA pH 8.0, 10 mM DTT) and stored at −80°C

### *In vitro* RNA-Dependent RNA Polymerase Reaction

The DNA fragment of pTNV(+)-PDS or pTNV(−)-PDS was amplified with primer pair 974/949 or 974/39 using pCB301-314-pTNV(+)-NbPDS-Rz or pCB301-314-pTNV(−)-NbPDS-Rz as the template. The resulted DNA was subjected to RNA synthesis by using T7 RNA polymerase (Takara, Cat. # 2540A). Briefly, a 100-μl mixture containing 10 μl 10 × T7 RNA polymerase buffer, 2 μl T7 RNA polymerase, 0.2 μl RNase inhibitors, 1 μg template DNA, 10 μl 0.1 M DTT, 10 μl 10 mM rNTP, and dH_2_O up to 100 μl was incubated at 42°C for 1.5 h. RNA was precipitated twice by isopropanol containing 10% (V/V) 10 M ammonium acetate and then washed with 70% ethanol.

The *in vitro* RNA-dependent RNA polymerase reaction was performed in a 50-μl reaction mix containing 50 mM Tris–HCl at pH 8.2, 10 mM MgCl_2_, 10 mM dithiothreitol, 100 mM potassium glutamate, 1.0 mM each ATP, CTP, and GTP, 0.01 mM UTP, 0.2 μl [32-P] UTP, 0.2 μl RNase inhibitor, 0.3 μg MBP-p82C, and 0.3 μg RNA transcripts. The reaction mix was incubated at 25°C for 2 h and terminated by the addition of 100 μl stop buffer (1% SDS, 0.05 M EDTA at pH 8.0). After extraction with water-saturated phenol-chloroform (1:1), precipitation in isopropanol containing 10% (V/V) 10 M ammonium acetate, and a washing step in 70% ethanol, the reaction product was mixed with loading buffer. The samples were either heated at 85°C for 5 min or kept on ice, and then analyzed by non-denaturing 5% polyacrylamide-8 M urea gel electrophoresis ran at 300 V for 6 h at 4°C. The gel was then imaged using a phosphorimager (Amersham Typhoon scanner IP, Cytiva).

## Data Availability Statement

The original contributions presented in the study are included in the article/supplementary material, further inquiries can be directed to the corresponding author/s.

## Author Contributions

KX initiated the project. WZ, YQ, LW, HZ, and KX designed the experiments. WZ, YQ, LZ, and JY conducted the experiments. WZ, YQ, LZ, LW, HZ, and KX analyzed the data. WZ and KX wrote the manuscript. All authors have read and approved the manuscript for publication.

## Conflict of Interest

The authors declare that the research was conducted in the absence of any commercial or financial relationships that could be construed as a potential conflict of interest.

## Publisher’s Note

All claims expressed in this article are solely those of the authors and do not necessarily represent those of their affiliated organizations, or those of the publisher, the editors and the reviewers. Any product that may be evaluated in this article, or claim that may be made by its manufacturer, is not guaranteed or endorsed by the publisher.
